# Effects of socio-economic household characteristics on traditional knowledge and usage of wild yams and medicinal plants in the Mahafaly region of south-western Madagascar

**DOI:** 10.1186/1746-4269-10-82

**Published:** 2014-12-30

**Authors:** Jessica N Andriamparany, Katja Brinkmann, Vololoniaina Jeannoda, Andreas Buerkert

**Affiliations:** Organic Plant Production and Agroecosystems Research in the Tropics and Subtropics, University of Kassel, Witzenhausen, Germany; Department of Biology and Vegetation Ecology, University of Antananarivo, Antananarivo, Madagascar

**Keywords:** Discriminant analysis, Local knowledge, Medicinal plants, Socio-economic factors, Wild yams

## Abstract

**Background:**

Rural households in the Mahafaly region of semi-arid SW-Madagascar strongly depend on the exploitation of natural resources for their basic needs and income regeneration. An overuse of such resources threatens the natural environment and people’s livelihood. Our study focuses on the diversity and use of wild yams and medicinal plants.

**Methods:**

We hypothesized that knowledge on the use of these resources highly depends on farmers’ socio-economic household characteristics. To test this hypothesis, an ethnobotanical survey was conducted based on semi-structured interviews recording socio-economic base data and information on local knowledge of medicinal and wild yam species. This was followed by field inventories compiling plant material for botanical identification.

**Results:**

Six species of wild yam and a total of 214 medicinal plants from 68 families and 163 genera were identified. Cluster and discriminant analysis yielded two groups of households with different wealth status characterized by differences in livestock numbers, off-farm activities, agricultural land and harvests. A generalized linear model highlighted that economic factors significantly affect the collection of wild yams, whereas the use of medicinal plants depends to a higher degree on socio-cultural factors.

**Conclusions:**

Wild yams play an important role in local food security in the Mahafaly region, especially for poor farmers, and medicinal plants are a primary source of health care for the majority of local people. Our results indicate the influence of socio-economic household characteristics on the use of forest products and its intensity, which should be considered in future management plans for local and regional forest conservation.

## Background

Madagascar constitutes one of the most important biodiversity hotspots worldwide with more than 90% of its plant and animal species being endemic, however, these resources are severely threatened by ecosystem degradation [1, 2]. With a gross national income (GNI) per capita of $828 [3], Madagascar ranks 151 out of 187 countries on the Human Development Index (HDI). Altogether, 74% of the population lives in rural areas of which 78% are considered poor [4] and mostly depend on the direct exploitation of natural resources (fields, water, forests) for their livelihoods.

The arid south-western region of Madagascar, commonly referred to as the Mahafaly region, is the country’s economically and climatically most disadvantaged area. It is characterised by high biotic endemism, listed as one of the 200 most important ecological regions in the world [5]. The subsistence production of the rural population comprises fishery, agriculture, livestock husbandry, and the collection of forest resources. Farmers’ livelihoods and economic development is hampered by a low level of education, limited income alternatives and poor infrastructure. The productivity of the cropland is limited by highly unpredictable rainfall and soil fertility constraints very similar to those encountered in the West African Sahel [6, 7]. Therefore, collection of forest products provides an important supplementary source of income [8], and an overuse of such resources threatens people’s livelihood. Among these forest products, the collection of wild yam (*Dioscorea* spp.) species and medicinal plants were identified as important for the local population [8, 9], as they contribute to the well-being of rural households in terms of direct use, human nutrition and income generation.

Medicinal plants constitute an important alternative to conventional medicine, especially for poor communities in rural areas without access to health services and they display a very large diversity in terms of species number [10]. According to the World Health Organization, approximately 80% of the world’s inhabitants rely predominantly on traditional medicine for their primary health care [11]. Of approximately 13,000 species present in Madagascar, about 3,500 are reported to have medicinal properties [12]. Madagascar has also a rich diversity of yam with altogether 40 species of which 27 are endemic and most of them have edible tubers [13], which are a staple food in many tropical countries. Wild yams have been reported to play an important role in rural household livelihoods system where they are traditionally eaten during periods of food insecurity [14]. The genus *Dioscorea* is distributed in various areas in Madagascar, but 24 species including 20 endemics were observed in the south western region [15]. These species are all edible, but the intensity of local usage depends on taste, local needs, market prices, location and harvested amounts. Other factors governing tuber use are differences in culture, gender, language, ethnicity, political belief system, personal preferences, appropriation skills and the availability of these resources in collection areas [16].

Detailed information on the importance of wild yams and medicinal plants for people’s livelihood and the factors influencing the intensity of their use are urgently required for natural resource management policy and planning and is lacking for SW-Madagascar. Therefore, the objective of this study was to analyse the diversity and use of wild yams and medicinal plants in the Mahafaly region, and to identify their role in the livelihoods of local people. We hypothesized that local knowledge on the usage of wild yams and medicinal plants depends on the socio-economic conditions and wealth status of households. Thereby, poorer households depend to a higher degree on forest resources and have a higher knowledge on their use than well-off farmers.

## Materials and methods

### Description of the study area

**Figure 1:**
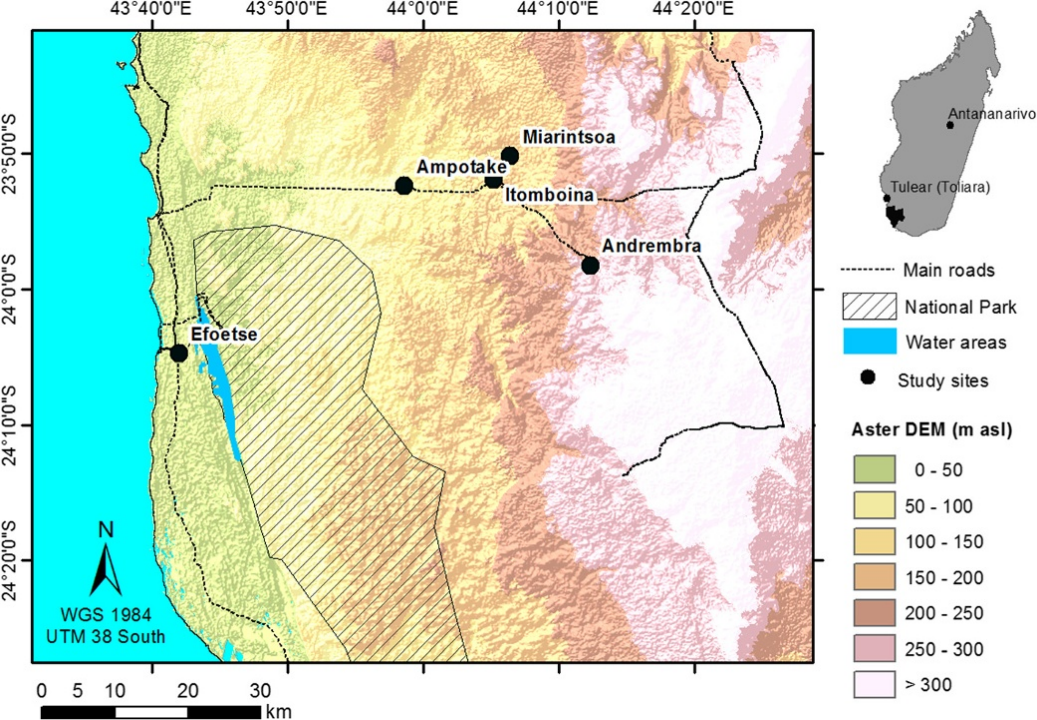
**Location of the study area in the Mahafaly region of SW-Madagascar.**

The study area is situated in the northern part of the Mahafaly region. The studied villages are located on the adjacent coast (littoral) and on the west side (plateau) of the Tsimanampetsotsa National Park (24°03′-24°12′S, 43°46′-43°50′E; Figure 1). The area is characterized by a dry and spiny forest vegetation with the highest level of endemism in plant species registered in Madagascar (48% of genera and 95% of species; [17]). The natural vegetation consists of a deciduous forest characterized by drought tolerant woody species of Didieraceae and Euphorbiaceae, xerophytic bushland and savannah. In the littoral zone dry forests on sandy soil dominate while on the plateau dry and spiny forests on tertiary limestone or ferruginous soil occur [18]. The semi-arid climate is characterized by an annual mean temperature of 24°C and a highly variable annual rainfall ranging between 300–350 mm in the littoral and 400-450 mm on the plateau [19]. The dry season lasts nine to ten months and the rainy season five months from November to April. The unreliability and unpredictability of rainfall is one of the major factors limiting agricultural production by the predominantly small holder farmers and herders, which partly rely on forest products to fulfil their daily needs throughout the year. During the past 40 years forest cover declined by 45% due to slash and burn agriculture and uncontrolled bushfires [20, 21]. In addition, the region has the lowest education rate of Madagascar and the majority of the households were classified as poor [22] in combination with a lack of basic health services and infrastructure. Altogether, 41% of the local population on the Mahafaly region is affected by food insecurity and famine [23]. Rapid population growth and the recent expansion of the Tsimanampetsotsa National Park (from 42,200 to 203,000 ha in 2007) have increased the pressure on the forests resources in and outside the park area [21, 24, 25]. Combined with the effects of climate change this leads to an increasing over-use of the natural resources in the Mahafaly region.

In the Mahafaly region wild yams are used to supplement cassava (*Manihot esculanta* Krantz) and maize (*Zea mays* L.), especially during hunger periods (‘Kere’). Local reports indicate that during the past years the amount of harvested wild yam tubers has strongly increased given a rising insufficiency of crop production.

### Field survey

The field work was conducted from June to December 2012 in five villages that were part of a larger village and household survey [21, 26]: (1) Efoetse in the littoral (S 24°4′42,41″- E 43°41′54,78″), (2) Ampotake (S 23°52′27,78″- E 43°58′36,55″), (3) Andremba (S 23°58′17,60″- E 44°12′17,05″), (4) Itomboina (S 23°51′59,15″- E 44°5′10,9″) and (5) Miarintsoa (S 23°50′14,21″- E 44°6′17,68″) on the plateau. Village selection was based on (1) market accessibility, (2) distance to the national park, (3) intensity of forest product collection of village inhabitants and (4) diversity of household activities. For each village, 50 households (HH) were randomly selected based on a complete household list (total N = 250). Pre-testing interviews and field observations were performed with key informants selected by snowball sampling [27]. Semi-structured interviews [28] were conducted with the household head after we received his consent. The Code of Ethics of the International Society of Ethnobiology was followed. If household head disagreed to take part in an interview, an alternative household was chosen based on an existing household list of the village. The questionnaire was divided in three thematic sections: (1) Information on socio-cultural and economic characteristic (family size, source of income, agricultural harvest, origin of the head and spouse, land area available for cultivation, livestock owned, harvest satisfaction, education level, ethnic group, religion, gender affiliation and age of respondents); (2) Household consumption, collection and use of wild yam species; (3) Medicinal plants and the knowledge about their uses. Respondents were also asked about specific plant parts used and the habitat from which they collected the plant material. All interviews were supplemented with field observations and forest walks. Since informants were only able to mention the local species name, plant specimen were collected in the field to establish a digital herbarium of inventoried specimens for botanical identification [29] in the Herbarium of the Botanical and Zoological Parc of Tsimbazaza (PBZT) in Antananarivo (Madagascar), following the nomenclature of the Tropicos database of the Missouri Botanical Gardens [30].

In the absence of any formal ethics committee the concept, content and questions related to this study conducted within the participatory SuLaMa (sustainable Land Management in South-Western Madagascar) project (http://www.sulama.de) were discussed and approved at the governmental and the village level in several meetings as were the outcomes of the interviews.

### Data analysis

The consumption, collection intensity and usage of wild yams were analysed using the following interview data: number of species collected, frequency of collection per month, period of collection per year, average number of tubers collected per collection event (estimated by the number of harvest holes), number of collectors per households, type of consumption (staple or additional food) and sale of tubers. The types of medicinal usage were categorized in different medicinal categories according to Cook [31]. To estimate the informant knowledge on the use of medicinal plants, the diversity of medicinal plant uses [32, 33] was calculated for each informant. The species (UV_S_) and the family use values (FUV) were computed (Table 1) [34, 35] to compare the importance of plant species and families.

**Table 1:** **Ethnobotanical indices used for measuring informant’s medicinal plant knowledge in the Mahafaly region of SW-Madagascar**

Indices	Calculation	Description
**Diversity of medicinal plant use (D)**	D = 1/∑ Pi^2^, where Pi^2^ is equal to the number of times a species was mentioned by informant ‘i’ divided by the total number of informants answer.	Simpson’s Reciprocal Index [32], adapted by [33]. Measures how many medicinal plant species an informant uses and how evenly his uses are distributed among the species.
**Species use value (UV**_**S**_ **)**	UV_S_ = ∑ UV_is_/n_i_, where UV_is_ is the sum of the total number of use citations by all informants for a given species and ni is the total number of informants.	Evaluates the relative importance of each plant species based on its relative use among informants [34], adapted by [35].
**Family use value (FUV)**	FUV = ∑UV_s_/n_s_, where ∑UV_S_ is the sum of species use value (UVs) within a family and n_s_ the number of species within a family.	Evaluates the use importance of a given plant family [34].

All statistical analyses were carried out using SPSS 17.0. A two-step cluster analysis was used to identify household groups based on socio-economic characteristics and plant use patterns. The existence of collinearity was tested based on correlation coefficients and suspicious data was removed from the dataset resulting in the following parameters used for cluster analysis: Education level, agricultural harvest, household activities, family size, tropical livestock units, agricultural area, medicinal plants used, number of medicinal uses and diversity of medicinal plant use (D), wild yam species collected, amount of tubers harvested (number of holes harvested for each collection), frequency of collection, sale, collection period and use of wild yams.

To evaluate the contribution of each variable in separating the resulting households groups, a Discriminant Analysis (DA) was conducted using the standardized canonical coefficients, canonical correlation coefficients, Eigen value and Wilk’s Lambda. A structure coefficient matrix was established which allowed to assess the importance of each variable in relation to the discriminant function.

A One Way ANOVA (Analysis of variance) was performed to compare the differences of knowledge and use between communities in relation to their location (villages). Additionally, we used Jaccard’s similarity index, which was based on species usage data to determine the similarity of species usage among villages [36].

To determine which cultural and socio-economic variables influence the use intensity and knowledge on medicinal plants and wild yams (response variables), we used a Generalized Linear Model (GLM) based on a Poisson distribution. The GLM consisted of two models with eight response variables, which explain the relationship between predictors and the knowledge on medicinal plants (number of medicinal plants used) and the use of wild yams (frequency of yam collection per month). The performance and the fit of the models were assessed using the Akaike Information Criterion (AIC; [37]). In each model, we only included main effects and choose the Type III analyses and Wald chi-square as statistical tests. The 0.05 significance level was used to assess if an independent variable related significantly to a dependent variable.

## Results and discussions

### Socio-economic characteristics of the interviewed households

Average household size varied between 6.3 persons in Itomboina and 7.2 persons in Miarintsoa (Table 2) whereby big households typically comprised a polygamous household head. Thus, each sub-family might live separately, but all family members eat together and share the same income. The education level of the households was highly variable across the villages, but in general, 30% of interviewed households did not receive formal education and only half visited at least the first year of primary school. The village with the highest rate of illiteracy, Ampotake, had no school. However, in Efoetse, where public and even private schools are available, literacy was high. The majority of the households comprise small holder farmers, which conduct different off-farm activities for cash income generation, such as salaried work, artisanal activities, trading, fishing, charcoal production or the collection of wood and other forest resources. The average household’s agricultural area was 2.2 ha of which some was partly left uncultivated due to heavy weed encroachment or a perceived decline in soil productivity. For the majority of households, periods of food insecurity due to unpredictable and insufficient rainfall are frequent and people heavily depend on supplementary off-farm income. Most of the household heads were born in the village where they live, only 26% are immigrants. The majority of households (60%) has traditional religious beliefs (ancestor reverence) and conduct ritual practices, while 30% are Christian (Catholic, Protestant or Anglicans).

**Table 2:** **Socioeconomic characteristics of the interviewed households (HH) in the five villages of the Mahafaly region in SW-Madagascar**

Characteristics		Ampotaka (n = 55)	Andremba (n = 50)	Itomboina (n = 50)	Miarintsoa (n = 50)	Efoetse (n = 50)	Total
**Age of the respondents**		41.7±17.3	44.2±15.5	46.7±18.3	40.4±17.6	42.6±19.9	43.1±17.8
**Family size**		6.8±3.9	6.4±3	6.3±3.3	7.2±3.7	6.7±2.3	6.7±3.3
**TLU**		1.6±3.1	5.1±9.2	4.8±7.5	6.9±10.9	9.2±12.8	5.5±9.5
**Land owned (ha)**		1.6±1.4	1.7±1.1	2.3±2.1	2.7±2.1	2.7±2.1	2.2±1.8
**Agricultural harvest (%)**	Low	44	36	62	32	14	38.0
	Medium	50	42	36	52	66	49.2
	High	6	20	2	16	20	12.8
**HH activities (%)**	Low	42	38	38	24	46	37.6
	Medium	36	46	44	46	40	42.4
	High	22	16	18	30	14	20.0
**Education level**	Low	52	22	32	16	24	29.2
	Visit primary school	34	56	50	54	54	49.6
	Finish primary school	14	22	18	30	22	21.2
**Origin of the head of the HH (%)**	Born in the village	*28*	10	40	38	18	26.8
	Not born in the village	*72*	90	60	62	82	73.2
**Gender of the respondent (%)**	Male	60	70	64	74	84	70.4
	Female	40	30	36	26	16	29.6
**Religion (%)**	No religion	14	8	4	6	17	9.7
	Traditional	60	62	64	58	55.3	59.9
	Christian	26	30	32	36	27.7	30.4

### Diversity and traditional use of plants

#### Wild yams

Altogether, six endemic species of wild yam were identified as potential food resource in the Mahafaly region: *Dioscorea ovinala* Baker (local name: ‘Angily’), *Dioscorea alatipes* Burk. & H. Perr. (‘Ovy’), *Dioscorea nako* H. Perr. (‘Fandra’), *Dioscorea fandra* H. Perr. (‘Andraha’), *Dioscorea bemandry* Jum. & H. Perr. (‘Baboky’) and *Dioscorea soso* Jum. & H. Perr. (‘Sosa’). Two thirds of the interviewed households (70%) were collecting wild yams. Yam collection was only uncommon in Efoetse where yams could be purchased from nearby markets. This is mainly due to the limited access to forest and yam resources in the littoral zone, where larger forest areas are lacking except of the Tsimanampetsotsa National Park area. In addition, wild yam species are relatively rare on the adjacent side of the national park where only *D. nako* occurs.

Wild yam tubers are used as a staple food by 42% of the households where they substitute cassava, maize or sweet potato (*Ipomoea batatas* L.), especially in villages situated near forest areas, where daily plant collection is possible. Respondents mentioned that they eat yams before the meal to reduce the quantity of staple food during the lean season. *D. alatipes* was most frequently collected (99% of yams collecting households), mainly because of its sweet taste and nutritional value. The so called water yam, *D. bemandry,* was also important and collected by 88% of households, because of its sweet taste and its big and long tubers (50–120 cm long). *D. soso* had the lowest collection rate (34% of households) given its scarce occurrence in the surrounding forests, although its taste is also appreciated by the local population.

#### Medicinal plants

**Figure 2:**
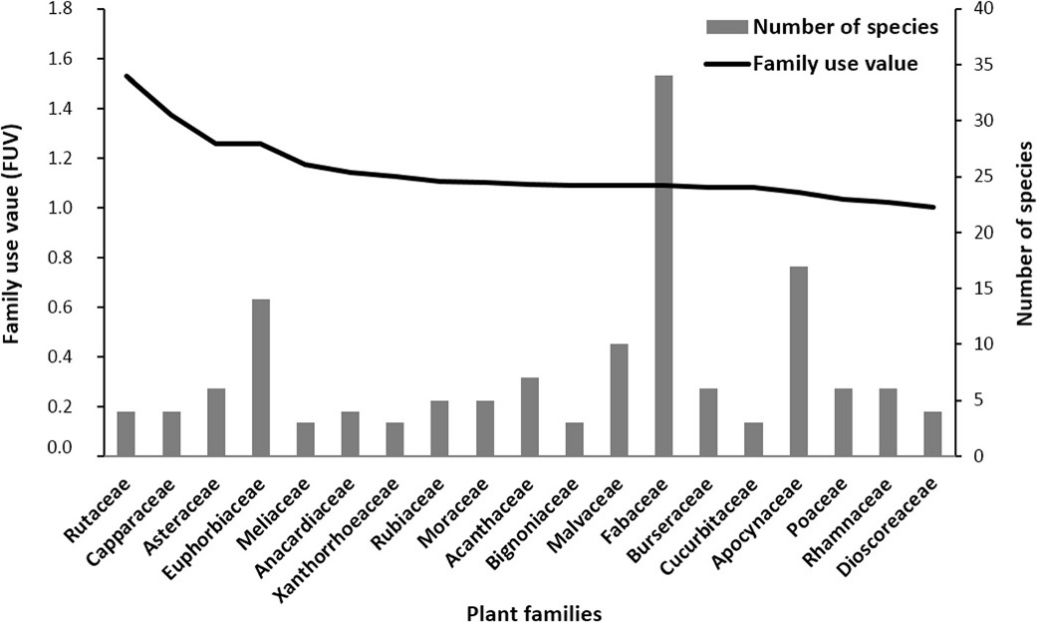
**Most important plant families identified by family use value (FUV, description see Table** 1 **) and number of medicinal plant species per family used in the Mahafaly region in SW-Madagascar.**

Altogether, 221 medicinal plants are used by the local people in the Mahafaly region (Table 3) of which 214 plant species were taxonomically identified and belong to 163 genera in 68 plant families. These plants are used to treat 46 diseases of human and livestock. Most species belonged to the Fabaceae (34 species), followed by Apocynaceae (17 species), Euphorbiaceae (16 species) and Malvaceae (10 species; Figure 2). Some families, such as the Aizoaceae, Aristolochiaceae, Flacourtiaceae, Myrtaceae, Sapotaceae, and Moringaceae were represented by only one species. Plant families with the highest FUV are Rutaceae (1.53), Capparaceae (1.37), Hernandiaceae (1.27) and Asteraceae (1.24). Among the 46 uses reported, the most common are digestive disorders, muscular skeletal problems and cosmetic care for women.

**Figure 3:**
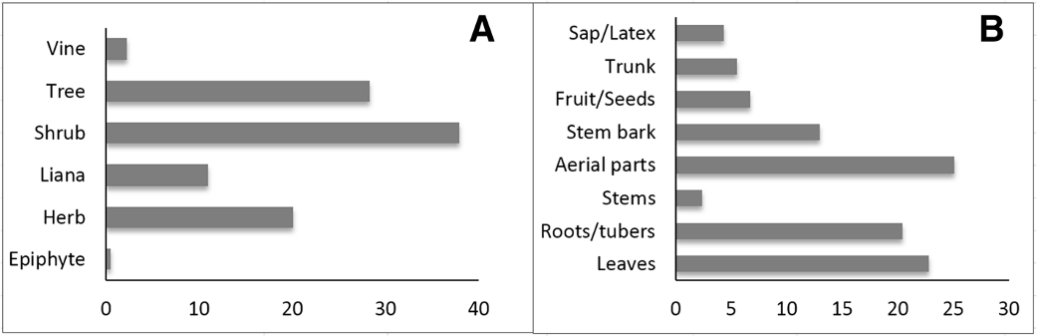
**Proportion of life forms used as medicinal plants (A); Proportion of plant parts used for traditional healing (B) in the Mahafaly region of SW-Madagascar.**

The growth forms of the recorded plants species are shrubs (38%), trees (28%), herbs (20%), lianas (11%), vines (2%), and epiphytes (less than 1%; Figure 3A). Most medicinal plants (82%) are collected in forest areas, 14% are cultivated and the rest is typically found in fallow land or rangelands such as bushland and grassland. Although the majority of the used plants are endemic to Madagascar (68%), exotic plants or plants that have a large worldwide distribution are used as well. Altogether, 95% of the recorded medicinal plants can be found in the Mahafaly region, the remainder are species bought or imported from the nearest town or from neighbouring regions.

**Table 3:** **List of medicinal plants species used in the Mahafaly region, SW-Madagascar**

Scientific name	Family	Local name	Use value	Citation (%)	Habitat	Parts used	Voucher number*
*Cedrelopsis grevei* Baill.	Rutaceae	Katrafay	3.06	99.6	Forest	Lv,Br,Tr	R. Rabevohitra 2390
*Croton* sp. 6	Euphorbiaceae	Tambio	3	0.4	Forest	Sb	-
*Boscia tenuifolia* A. Chev.	Capparaceae	Lalangy	2	0.4	Forest	Ar	-
*Pluchea grevei* (Baill.) Humbert	Asteraceae	Samonty	1.91	5.5	Forest	Lv	J.Bosser 9917
*Aloe divaricata* A. Berger	Xanthorrhoeaceae	Vahondrandro	1.87	100	Forest	Lx	Reynold 7860
*Cadaba virgata* Bojer	Capparaceae	Tsihariharinaliotse	1.5	0.9	Forest	Ar	Bewerley Lewis 534
*Tamarindus indica* L.	Fabaceae	Kily	1.47	59.2	Forest, Fallow	Lv,Br,Fr	Thomas B. Croat 31108
*Neobeguea mahafaliensis* Leroy, Jean F. P.	Meliaceae	Handy	1.44	91.1	Forest	Sb,Tr	R. Decary 16206
*Croton* sp. 4	Euphorbiaceae	Zalazala	1.38	14.5	Forest	Br	-
*Ficus lutea* Vahl.	Moraceae	Amonta	1.38	6.8	Forest	Ar	G McPherson 14634
*Psiadia angustifolia* (Humbert) Humbert	Asteraceae	Ringandringa	1.38	22.1	Forest	Lv	RN 3806
*Sida rhombifolia* L.	Malvaceae	Mandravasarotse	1.38	6.8	Fallow	Ar	Thomas B. Descoings 30725
*Croton geayi* Leandri	Euphorbiaceae	Pisopiso	1.36	72.3	Forest	Sb,Br	H. Humbert 2397
*Lemuropisum edule* H. Perrier	Fabaceae	Berotse	1.36	10.6	Forest	Sb	J. Bosser 1984
*Acacia sakalava* Drake	Fabaceae	Roymena	1.33	1.3	Savanna, Forest	Ar	J.F. Villiers 4056
*Dalbergia* sp.	Fabaceae	Manary	1.33	12.8	Forest	Br	-
*Acacia bellula* Drake	Fabaceae	Rohy	1.3	14	Forest	Ar	R. Ranaivojaona 492
*Hernandia voyronii* Jum.	Hernandiaceae	Hazomalany	1.3	4.3	Forest	Tr	J.Bosser 9178
*Euphorbia tirucalli* L.	Euphorbiaceae	Laro	1.29	53.6	Forest	Lv,St	P.B. Phillipson 2480
*Coffea grevei* Drake ex A.Chev	Rubiaceae	Hazombalala	1.28	31.5	Forest	Sb,Ar	C.C.H. Jonngkind 3746
*Aloe vaombe* Decorse & Poisson	Xanthorrhoeaceae	Vahombe	1.25	37.9	Forest	Lx	H. Humbert 5418
*Cynanchum mahafalense* Jum. & H. Perrier	Apocynaceae	Vahimasy	1.25	19.2	Forest	Sb,St	B. Descoings 3251
*Citrullus lanatus* (Thunb.) Mansf. & Naka	Cucurbitaceae	Voamanga	1.24	20.9	Crop field	Ar	J. Bosser 13567
*Croton kimosorum* Leandri	Euphorbiaceae	Zanompoly	1.24	26.8	Forest	Br	J. Bosser 10429
*Gyrocarpus americanus* Jacq.	Hernandiaceae	Kapaipoty	1.24	10.6	Forest	Lv	P.B. Phillipson 2350
*Operculicarya decaryi* H. Perrier	Anacardiaceae	Jabihy	1.24	52.3	Forest	Br,Tr	P. Morat 696
*Tetrapterocarpon geayi* Humbert	Fabaceae	Hazolava/Voaovy	1.24	38.7	Forest	Sb,Br	B. Descoings 1433
*Erythroxylum retusum* Baill. ex O.E. Schulz	Erythroxylaceae	Montso	1.23	71.9	Forest	Lv	P.B. Phillipson 2464
*Mangifera indica* L.	Anacardiaceae	Mangavato	1.23	4.7	Crop field	Br	_
*Polycline proteiformis* Humbert	Asteraceae	Zira	1.22	3.4	Forest	Sb,ar	J. Bosser 248
*Leptadenia madagascariensis* Decne.	Apocynaceae	Taritarika/Mozy	1.21	46.4	Forest	Sb,Ar	B. Descoings 1243
*Ruellia anaticollis* Benoist	Acanthaceae	Reforefo	1.21	7.2	Forest	Ar	P.B.Phillipson 1795
*Bulbostylis xerophila* H. Cherm.	Cyperaceae	Foentany	1.2	2.1	Forest	Ar	M.R. Decary 8531
*Grewia* sp.	Malvaceae	Malimatse	1.2	2.1	Forest	Br	-
*Mundulea* sp. 1	Fabaceae	Sofasofa	1.2	6.4	Forest	Ar	-
*Oeceoclades decaryana* (H. Perrier) Garay & P. Taylor	Orchidaceae	Hatompototse	1.2	2.1	Forest	St	Gordon Mc Pherson 17376
*Paederia grandidieri* Drake	Rubiaceae	Tamboro	1.19	11.1	Forest	Lv	P.B. Phillipson 2810
*Salvadora angustifolia* Turill	Salvadoraceae	Sasavy	1.19	79.6	Forest	Lv,Sb	P.B. Phillipson 3711
*Vanilla madagascariensis* Rolfe	Orchidaceae	Amalo	1.19	8.1	Forest	St	-
*Aristolochia acuminate* Lamk.	Aristolochiaceae	Totonga	1.18	41.3	Forest	Sb	P. Morat 3512
*Commiphora lamii* H. Perrier	Burseraceae	Holidaro	1.17	5.1	Forest	Br	C.C.H. Jongkind 3681
*Cassia siamea* Lam.	Fabaceae	Farefare	1.16	21.3	Forest	Br	M. B. Dupuy M98
*Didierea madagascariensis* Baill.	Didieraceae	Sono	1.16	12.8	Forest	Tr	D. Lorence 1928
*Securinega perrieri* Leandri	Phyllanthaceae	Hazomena	1.16	10.6	Forest	Lv	Herb., Inst.Sci. Mad. 4497
*Commiphora mahafaliensis* Capuron	Burseraceae	Maroampotony	1.15	8.5	Forest	Ar	-
*Cynanchum grandidieri* Liede & Meve	Apocynaceae	Betondro	1.15	24.7	Forest	Sb	-
*Indigofera compressa* Lam.	Fabaceae	Hazomby	1.15	36.6	Forest	Ar	M.R. Decary 9147
*Ipomoea pes-caprae* (L.) R. Br*.*	Convolvulaceae	Fobo	1.15	8.5	Seaside	Sb	Robert W. Books 19
*Solanum hippophaenoïdes* Bitt.	Solanaceae	Hazonosy	1.15	25.5	Forest	Lv,Sb	-
*Croton* sp. 5	Euphorbiaceae	Andriambolafotsy	1.14	3	Forest	Lv	-
*Mundulea* sp. 2	Fabaceae	Taivosotse	1.14	3	Forest	Ar	-
*Zygophyllum depauperatum* Drake	Zygophyllaceae	Filatatao	1.14	3	Forest	Lv	J. Bosser 10129
*Blepharis calcitrapa* Benoist	Acanthaceae	Sitsitse	1.13	19.6	Forest	Sb	H. Humbert 5136
*Commiphora monstruosa* (H. Perrier) Capuron	Burseraceae	Taraby	1.13	19.2	Forest	Ar,Tr	-
*Cynanchum perrieri* Choux	Apocynaceae	Ranga	1.13	66.8	Forest	St	Labat J-N 2414
*Henonia scoparia* Moq.	Amaranthaceae	Fofotse	1.13	10.2	Forest	Lv	M.R. Decary 2531
*Hypoestes phyllostachya* Baker	Acanthaceae	Fotivovona	1.13	13.6	Forest	Ar	J. Bosser 43
*Indigofera mouroundavensis* Baill.	Fabaceae	Sambobohitse	1.13	3.4	Forest	Sb	Jacqueline & M. Peltier 3171
*Opuntia* sp. 2	Cactaceae	Raketamena	1.13	6.4	Crop field, Fallow	Sb	-
*Stereospermum nematocarpum* DC.	Bignoniaceae	Mahafangalitse	1.13	23.4	Forest	Br	Herb. Inst. Sci. Mad. 4630
*Streblus* sp.	Moraceae	Hazondranaty	1.13	20.4	Forest	Sb.Tr	
Zea mays L.	Poaceae	Tsako	1.13	6.4	Crop field	Fr	-
*Ziziphus spina-christi* (L.) Willd.	Rhamnaceae	Tsinefo	1.13	34.5	Crop field, Fallow	Br	J. Bosser 416
*Euphorbia stenoclada Baill.*	Euphorbiaceae	Samata	1.12	28.9	Forest	Lv,Sb	RN 4768
*Grewia leucophylla* Capuron	Malvaceae	Fotilambo	1.12	7.2	Forest	Sb,Br	Michelle Sauther 23
*Rhigozum madagascariense* Drake	Bignoniaceae	Hazonta	1.12	17.9	Forest	Ar	J. Bosser 14420
*Grewia humblotii* Baill.	Malvaceae	Sely	1.11	26.4	Forest	Sb,Br	-
*Lasiocladus anthospermifolius* Bojer ex Nees	Acanthaceae	Maintemaso	1.11	24.3	Forest	Lv,Sb	J.N. Labat 2696
*Cajanus cajan* (L.) Millsp.	Fabaceae	Ambatry	1.1	15.3	Crop field	Ar	Thomas B. Croat 32106
*Cynanchum nodosu* (Jum. & H. Perrier) Desc.	Apocynaceae	Try	1.1	24.3	Forest	Sb	P.B. Phillipson 1671
*Adenia olaboensis* Claverie	Passifloraceae	Hola	1.09	4.7	Forest	Lx	Jacqueline & M. Peltier 1396
*Azima tetracantha* Lam.	Salvadoraceae	Tsingilo	1.09	9.4	Forest	Lv	M.R Decary 3470
*Hydnora esculenta* Jum. & H. Perrier	Hydnoraceae	Voantany	1.09	9.8	Forest	Sb	Herb., Inst.sci. Mad. 2
*Sclerocarya birrea* subsp. *caffra* (Sond.) Kokwaro	Anacardiaceae	Sakoa/Sakoamanga	1.09	38.7	Savana	Lv,Br	D.J. Mabberley 732
*Secamone tenuifolia* Decne.	Apocynaceae	Langolora	1.09	14.5	Forest	Sb	J. Bosser 17209
*Abutilon indicum* (L.)Sweet	Malvaceae	Lahiriky	1.08	22.1	Forest, Fallow	Ar	L.J. Dorr 4056
*Capuronianthus mahafalensis* J.-F. Leroy	Meliaceae	Ringitse	1.08	5.1	Forest	Sb	_
*Mollugo decandra* Scott-Elliot	Molluginaceae	Andriamanindry	1.08	10.2	Forest	Ar	H. Humbert 5293
*Moringa drouhardii* Jum.	Moringaceae	Maroserana	1.08	5.5	Forest	Ar	B. Descoings 2411
*Pentarhopalopilia madagascariensis* Cavaco & Keraudren	Opiliaceae	Fandriandambo	1.08	10.2	Forest	Ar	B. Descoings 1214
*Ximenia perrieri* Cavaco & Keraudren	Ximeniaceae	Kotro	1.08	26.8	Forest	Lv,Sb	Rauh 1221
*Cymbopogon excavatus* (Hochst.) Stapf ex Burtt Davy	Poaceae	Ahibero	1.07	1.7	Forest	Lv	Bosser 5208
*Avicennia marina* (Forssk.) Vierh.	Acanthaceae	Afiafy	1.06	3.8	Forest	Br	James L. Zarucchi 7552
*Enterospermum pruinosum* (Baill.) Dubard & Dop	Rubiaceae	Mantsake	1.06	7.2	Forest	Br	-
*Hyphaene sp.*	Arecaceae	Satra	1.06	22.1	Crop field	Lv,Sb	
*Zingiber officinale* Roscoe	Zingiberaceae	Sakaviro	1.06	14.5	Crop field	Sb	M.R. Decary 1440
*Chloroxylon falcatum* Capuron	Rutaceae	Mandakolahy	1.05	35.3	Forest	St	-
*Jatropha mahafalensis* Jum. & H.Perrier	Euphorbiaceae	katratra	1.05	46	Forest	Lv,Lx	H. Humbert 2521
*Pentatropis nivalis* subsp. *madagascariensis* (Decne.) Liede & Meve	Apocynaceae	Tinaikibo	1.05	61.7	Forest	Ar	-
*Agave sisalana* Perrine	Agavaceae	Lalohasy	1.04	19.6	Forest	Lx	-
*Commiphora simplicifolia* H. Perrier	Burseraceae	Sengatse	1.04	10.6	Forest	Ar	Z.S. Rogers 870
*Hippocratea angustipetala* H. Perrier	Celastraceae	Vahimpindy	1.04	11.1	Forest	Ar	-
*Musa* sp.	Musaceae	Kida	1.04	46.8	Crop field	Fr	-
*Pentopetia androsaemifolia* Decne.	Apocynaceae	Ntsompia	1.04	9.8	Crop field, Fallow	Lv	Arne Anderberg 123
*Strychnos* sp. 2	Loganiaceae	Mangerivorika	1.04	19.6	Forest	Ar	-
*Tridax procumbens* L.	Asteraceae	Angamay	1.04	53.6	Crop field, Fallow	Lv	P.B. Phillipson 1791
*Uncarina stellulifera* Humbert	Pedaliaceae	Farehitse	1.04	9.8	Forest	Lv	P.B. Phillipson 2723
*Delonix floribunda* (Baill.) Capuron	Fabaceae	Fengoky	1.03	40	Forest	Lx	J. Bosser 13584
*Jatropha curcas* L.	Euphorbiaceae	Savoa	1.03	39.2	Forest	Lv,Sb,Lx	P.B. Phillipson 1725
*Loeseneriella rubiginosa* (H. Perrier) N. Hallé	Celastraceae	Timbatse	1.03	35.7	Forest	Lv	B. Du puy MB 570
*Terminalia ulexoides* H. Perrier	Combretaceae	Fatra	1.03	13.6	Forest	Sb	L. J. Dorr 4057
*Androya decaryi* H.Perrier	Scrophulariaceae	Manateza	1.02	23	Forest	Lv	Herbier du Laboratoire de Botanique 1777
*Fernandoa madagascariensis* (Baker) A.H. Gentry	Bignoniaceae	Somontsoy	1.02	46.8	Forest	Lv,Br	L.J. Dorr 3960
*Ocimumcanum* Sims.	Lamiaceae	Romberombe	1.02	37.9	Forest	Ar	B. Croat 31282
*Tabernaemontana* sp.	Apocynaceae	Feka	1.01	40.4	Forest	Sb	-
*Zanthoxylum tsihanimposa* H.Perrier	Rutaceae	Manongo	1.01	60	Forest	Sb	P. Morat 4677
*Abrus precatorius* L.	Fabaceae	Voamena	1	2.6	Forest	Ar	J. Bosser 19395
*Acacia farnesiana* (L.) Willd.	Fabaceae	Kasy	1	1.7	Savanna	Ar	D.J. & B.P. Dupuy M69
*Acacia* sp. 5	Fabaceae	Anadrohy	1	0.4	Forest	Br	-
*Acacia viguieri* Villiers & Du Puy	Fabaceae	Roybenono	1	3	Forest	Ar	H. Humbert 2487
*Adansonia rubrostipa* Jum. & H.Perrier	Malvaceae	Fony	1	2.6	Forest	Fr	J. Bosser 15743
*Adansonia za* Baill.	Malvaceae	Zan	1	4.3	Forest	Fr	P.B. Phillipson 2638
*Aerva javanica* (Burm. f.) Juss.	Amaranthaceae	Volofoty	1	6	Forest	Sb	M.R. Decary 18863
*Alantsilodendron alluaudianum* (R.Vig.) Villiers	Fabaceae	Havoa	1	0.4	Forest	Ar	-
*Albizia bernieri* E. Fourn. ex Villiers	Fabaceae	Halimboro	1	2.1	Forest	Br	P.B. Phillipson 5285
*Albizia tulearensis* R.Vig.	Fabaceae	Mendoravy	1	0.4	Forest	Br	D.J. & B. P. Dupuy M54
*Allium sativum* L.	Amaryllidaceae	Tongologasy	1	5.5	Crop field	Sb	-
*Aloe antandroi* (R.Decary) H. Perrier	Xanthorrhoeaceae	Sotry	1	2.1	Forest	Lv	M.R. Decary 9886
*Alysicarpus vaginalis* (L.) D.C.	Fabaceae	Tokampototse	1	6.4	Crop field, Fallow	Ar	Thomas B. Croat 31195
*Amaranthus viridis* L.	Amaranthaceae	Beamena	1	0.4	Crop field, Fallow	Ar	-
*Anisotes madagascariensis* Benoist	Acanthaceae	*Hazontsoy*	1	1.3	Forest	Ar	Rauh 1097
*Arachis hypogaea* L.	Fabaceae	Kapiky	1	17.5	Crop field	Fr	-
*Asparagus calcicola* H. Perrier	Asparagaceae	Fio	1	0.4	Forest, Fallow	Sb	J. Bosser 10599
*Azadirachta indica* A. Juss.	Meliaceae	Nimo	1	6.4	Forest	Lv	Armand Rakotozafy 1798
*Barleria brevituba* Benoist	Acanthaceae	Patipatikantala	1	0.4	Savanna, Fallow	Ar	P. Morat 627
*Bathiorhamnus cryptophorus* Capuron	Rhamnaceae	Losy	1	11.5	Forest	Sb	-
*Berchemia discolor* (Klotzsch) Hemsl.	Rhamnaceae	Vorodoke	1	1.7	Forest	Ar	-
*Calopyxis grandidieri* (Drake) Capuron ex Stace	Combretaceae	Tsambara	1	1.7	Forest	Fr	B Lewis 1294
*Capsicum* sp.	Solanaceae	Sakay	1	21.3	Crop field	Fr	
*Capurodendron androyense* Aubrév.	Sapotaceae	Nato	1	11.5	Forest	Sb,Br	J. Bosser 10352
*Carica papaya* L.	Caricaceae	Papaye	1	6	Crop field	Lv	Herbier du Jardin Botanique 324
*Carissa spinarum* L.	Apocynaceae	Lamontindahy	1	0.4	Forest	Ar	-
*Chadsia grevei* Drake	Fabaceae	Sanganakoholahy	1	7.7	Forest	Ar	D.J. & B.P. Dupuy M38
*Chamaesyce hirta* (L.) Millsp.	Euphorbiaceae	Kimenamena	1	7.7	Crop field	Lv	Robert W. Brooks 8
*Citrus medica* L.	Rutaceae	Tsoha	1	0.4	Crop field	Sb	-
*Cocos nucifera* L.	Arecaceae	Voanio	1	0.4	Seaside	Fr	-
*Colvillea racemosa* Bojer	Fabaceae	Sarongaza	1	14	Forest	Br	P.B. Phillipson 2802
*Commiphora humbertii* H. Perrier	Burseraceae	Andrambely	1	0.4	Forest	Lv	S. Eboroke 870
*Commiphora marchandii* Engl.	Burseraceae	Vingovingo	1	0.4	Forest	Ar	James S. Miller 6160
*Cordia caffra* Sond.	Boraginaceae	Varo	1	1.7	Forest	Lv	Thomas B .Croat 30787
*Crinum asiaticum* L.	Amaryllidaceae	Tongolondolo	1	0.4	Forest	Sb	-
*Crotalaria androyensis* R. Vig*.*	Fabaceae	Katsankantsa	1	0.9	Forest	Ar	M.R. Decary 9517
*Crotalaria fiherenensis* R.Vig.	Fabaceae	Voniloha	1	0.9	Savanna, Forest, Fallow	Ar	_
*Croton catatii* Baill.	Euphorbiaceae	Somorombohitse	1	0.9	Forest	Ar	M.R. Decary 10495
*Cryptostegia madagascariensis* Bojer ex Decne	Apocynaceae	Lombiry	1	4.7	Forest	Lv,Sb	P.B. Phillipson 2622
*Cucurbita maxima* Duch.	Cucurbitaceae	Trehaky	1	0.4	Crop field	Ar	J.Bosser 13577
*Cymbopogon citratus* (DC.) Stapf	Poaceae	Veromanitse	1	0.4	Crop field	Ar	-
*Cynodon dactylon* (L.) Pers.	Poaceae	Kidresy	1	4.7	Forest	Ar	J. Bosser 10540
*Cyphostemma amplexicaule* Desc.	Vitaceae	Tahezantrandrake	1	1.3	Forest	Lv	J. Bosser 19194
*Dicoma incana* (Baker) O. Hoffm.	Asteraceae	Peha	1	10.2	Forest	Sb	P.B. Phillipson 2426
*Dicraeopetalum mahafaliense* (M.Pelt.) Yakovlev	Fabaceae	Lovainafy	1	1.7	Forest	Br	Thomas B. Croat 30969
*Dioscorea bemandry* Jum. & H. Perrier	Dioscoreaceae	Baboke	1	0.4	Forest	Sb	L.R. Caddick 339
*Dioscorea fandra* H. Perrier	Dioscoreaceae	Andraha	1	2.1	Forest	Sb	Gordon McPherson 17451
*Dioscorea nako* H. Perrier	Dioscoreaceae	Fandra	1	0.4	Forest	Sb	L.R. Caddick 331
*Dioscorea ovinala* Baker	Dioscoreaceae	Behandaliny	1	0.9	Forest	Ar	J.N. Labat 2111
*Diospyros tropophylla* (H. Perrier) G.E. Schatz & Lowry	Ebenaceae	Remeloky	1	2.1	Forest	Ar	P. Morat 565
*Ehretia decaryi* J. S. Mill.	Boraginaceae	Lampana	1	6	Forest	Ar	J. Bosser 10116
*Enterospermum madagascariense (*Baill.) Homolle	Rubiaceae	Masonjoany	1	0.4	Forest	Tr	-
*Erythrophysa aesculina* Baill.	Sapindaceae	Handimbohitse	1	2.6	Forest	Ar	G.E. Schatz 1777
*Euclinia suavissima* (Homolle ex Cavaco) J.-F. Leroy	Rubiaceae	Voafotaky	1	0.9	Forest	Fr	J. Bosser 13353
*Euphorbia arahaka* Poisson	Euphorbiaceae	Samatafoty	1	14.9	Savanna, Forest, crop field	Lv	M.D. Decary 3008
*Ficus polita* Vahl	Moraceae	Aviavy	1	3.8	Forest	Br	M.R. Decary 5031
*Ficus sp.*	Moraceae	Nonoka	1	1.7	Fallow, Forest	Br	-
*Ficus trichopoda* Baker	Moraceae	Fihamy	1	39.2	Forest	Tr	S.T. Malcomber 1116
*Flacourtia indica* (Burm. f.) Merr.	Salicaceae	Lamonty	1	3.8	Forest	Sb,Fr	C.C.H. Jongkind 3720
*Gnidia linearis* (Leandri) Z.S. Rogers	Thymeleaceae	Ronisa	1	1.3	Forest	Lv	Z.S. Rogers 930
*Gonocrypta grevei* (Baill.) Costantin & Gallaud	Apocynaceae	Piravola	1	6.8	Forest	Lx	P.B. Phillipson 1669
*Gossypium arboreum* L.	Malvaceae	Hasy	1	3.8	Crop field, Fallow	Lv	H. Humbert 5166
*Grewia grevei* Baillon	Malvaceae	Tombokampaha	1	0.9	Forest	Ar	J. Bosser 19338
*Grewia microcyclea* (Burret) Capuron & Mabb.	Malvaceae	Hazofoty	1	3.8	Forest	Br	Jacqueline & M. Peltier 1285
*Helinus integrifolius* (Lam.) Kuntze	Rhamnaceae	Masokarany	1	2.1	Forest	Ar	P.B. Phillipson 1737
*Indigofera tinctoria* L.	Fabaceae	Sarikapiky	1	49.4	Fallow, Savanna	Ar	J.N. Labat 2104
*Ipomea* sp. 1	Convolvulaceae	Sarivelahy	1	1.7	Forest, Savanna, Fallow	Lv	-
*Ipomea* sp. 2	Convolvulaceae	Velahy	1	1.3	Forest	Lx	-
*Kalanchoe beharensis* Drake	Crassulaceae	Mongy	1	0.4	Forest	Lv	James L. Zarucchi 7471
*Kalanchoe* sp.	Crassulaceae	Relefo	1	3.4	Forest	Lv	-
*Karomia microphylla* (Moldenke) R.B. Fern.	Lamiaceae	Forimbitika	1	0.9	Forest	Br	P.B. Phillipson 3438
*Kleinia madagascariensis* (Humbert) P. Hallyday	Asteraceae	Malaohira	1	2.6	Forest	Ar	P.B. Phillipson 2475
*Koehneria madagascariensis* (Baker) S.A. Graham, Tobe & Baas	Lythraceae	Fizolotsora	1	1.7	Forest	Ar	L.J. Dorr 4063
*Lablab purpureus* (L.) Sweet	Fabaceae	Antaky	1	9.4	Crop field	Fr	Michelle Sauther 27
*Leucosalpa grandiflora* Humbert	Orobanchaceae	Tamborisahy	1	1.7	Forest	Sb	P. Morat 2978
*Maerua filiformis* Drake	Capparaceae	Somangy	1	1.3	Forest	Lv,Ar	P.B. Phillipson 2417
*Maerua nuda* Scott-Elliot	Capparaceae	Somangilahy	1	1.7	Forest	Lv	J. Bosser 10507
*Manihot esculenta* Crantz	Euphorbiaceae	Balahazo	1	8.1	Crop field	Lv,Sb	-
*Margaritaria anomala* (Baill.) Fosberg	Phyllanthaceae	Tsivano	1	18.7	Forest	Sb	-
*Marsdenia cordifolia* Choux	Apocynaceae	Bokabe	1	2.6	Forest	Lx	P.B. Phillipson 2741
*Mundulea stenophylla* R. Vig.	Fabaceae	Rodrotse	1	1.7	Forest	Lv	M.R. Decary 2527
*Olax andronensis* Baker	Olacaceae	Bareraky	1	0.4	Forest	Sb	L.J. Razafintsalama 785
*Opuntia monacantha* Haw.	Cactaceae	Notsoky	1	2.6	Fallow, Savanna	Fr	-
*Pachypodium geayi* Costantin & Bois	Apocynaceae	Vontake	1	0.4	Forest	Tr	P.B Phillipson 2610
*Panicum pseudowoeltzkowii* A. Camus	Poaceae	Ahikitoto	1	0.4	Forest	Lv	J. Bosser 308
*Panicum* sp.	Poaceae	Mandavohita	1	0.4	Fallow, Forest, Savanna	Ar	-
*Persea americana* Mill.	Lauraceae	Zavoka	1	0.9	Crop field	Fr	_
*Pervillaea phillipsonii* Klack.	Apocynaceae	Sangisangy	1	0.4	Forest	Ar	P.B. Phillipson 3472
*Phaseolus lunatus* L.	Fabaceae	Kabaro	1	5.5	Crop field	Fr	J. Bosser 1011
*Phyllanthus casticum* Willemet	Phyllanthaceae	Sanira	1	6	Forest	Lv	P.B. Phillipson 2392
*Plumbago aphylla* Bojer ex Boiss.	Plumbaginaceae	Motemote	1	1.7	Forest	Ar	H. Humbert 19960
*Poupartia minor* (Bojer) L. Marchand	Anacardiaceae	Sakoakomoky	1	2.1	Forest	Br	P.B. Phillipson 1813
*Psidium* sp.	Myrtaceae	Goavy	1	0.4	Crop field, Fallow	Lv	-
*Radamaea montana* Benth.	Orobanchaceae	Tamotamo	1	31.5	Forest	Sb	J. Bosser 6071
*Rhopalopilia hallei* Villiers	Opiliaceae	Malainevotsy	1	11.5	Forest	Ar	-
*Ricinus communis* L.	Euphorbiaceae	Kinana	1	5.5	Crop field, Fallow	Lv	Thomas B. Croat 28615
*Roupellina boivinii* (Baill.) Pichon	Apocynaceae	Lalondo	1	0.9	Forest	Lv	-
*Secamone geayi* Costantin & Gallaud	Apocynaceae	Kililo	1	4.7	Forest	Ar	J. Bosser 15917
*Strychnos madagascariensis* Poir.	Loganiaceae	Bakoa	1	7.7	Forest	Sb,Fr	J. Bosser 14492
*Tephrosia purpurea* (L.) Pers.	Fabaceae	Engetsengetse	1	5.1	Forest	Lv	Jacqueline & M. Peltier 9936
*Terminalia disjuncta* H. Perrier	Combretaceae	Taly	1	1.7	Forest	Ar	B. Dupuy 629
*Trema orientalis* (L.) Blume	Cannabaceae	Andrarezona	1	0.4	Forest	Tr	B. Lewis 1292
*Typha angustifolia* L.	Typhaceae	Vondro	1	0.4	Forest	Lv	M.R. Decary 14868
*Vigna unguiculata* (L.) Walp.	Fabaceae	Loji	1	20.4	Crop field	Fr	Thomas B. Croat 32050
*Xerophyta tulearensis* (H. Perrier) Phillipson & Lowry	Velloziaceae	Tsimatefaosa	1	0.4	Forest	Ar	P.B Phillipson 2459
*Xerosicyos danguyi* Humbert	Cucurbitaceae	Tapisaky	1	1.3	Forest	Lv	Thomas B. Croat 30795
*Ziziphus mauritiana* Lam.	Rhamnaceae	Konazy	1	0.4	Savanna	Br	D. Seigler 12891
*Ziziphus mucronata* Willd.	Rhamnaceae	Tsinefonala	1	4.7	Forest	Br	Harb. Inst. Sci. Mad. 4517

Lv = Leaves, Ar = Aerial parts, Sb = Subterranean parts, Fr = Fruits or seeds, Lx = Sap or latex, Tr= Trunk, St = Stems, Br =stem barks; (*) Voucher number represents the number of the specimens from which our plants were determined in Tsimbazaza Herbarium, Madagascar.

The most frequently collected plant parts are the aboveground plant material (i.e., stems and leaves, 25%), leaves (23%) and subterranean parts (roots and tubers, 20%; Figure 3B). Single stems are not often used for medicinal purposes (2%), whereas the roots of plants are used, especially for post-delivery treatment, women genital and cosmetic care, such as *Ximenia perrieri* (‘Kotro’). Sometimes people use different parts of the same plant, especially if it has a high use value (i.e. used for different medicinal purposes), such as *Neobeguea mahafaliensis* (‘Handy’). The stem barks of this species are used to treat muscular-skeletal problems and its below ground parts serve women during the post-delivery process.

Regarding the use of species, *Aloe divaricata* (used by 100% of informants), *Cedrelopsis grevei* (100%) and *Neobeguea mahafaliensis* (91%) predominate. *Aloe divaricata* is a locally important species with 28 different uses. Altogether, 46 types of medicinal uses were reported (Cook [31]; Table 4). Some species, such as *Operculicarya decaryi,* may also be used in multiple ways such as a body tonic, for women genital care and to alleviate nutritional disorders during famine periods. *Tamarindus indica* was used to treat eye problems, but it is similarly important to alleviate nutritional disorders.

**Table 4:** **Categories of diseases and their respective most cited plant species in the Mahafaly region of SW Madagascar**

Diseases and use category	Most cited species
**Digestive disorders**	*Aloe divaricata* A. Berger*, Cedrelopsis grevei* Baill.
**Muscular_Skeletal**	*Neobeguea mahafaliensis* J.-F. Leroy*, Cedrelopsis grevei* Baill.
**Eye problems**	*Tamarindus indica* L.*, Jatropha mahafalensis* Jum. & H. Perrier*, Fernandoa madagascariensis* (Baker) A.H. Gentry
**Wound/Injury/Swelling**	*Tridax procumbens* L.*, Tabernaemontana sp.*, *Croton geayi* Leandri
**Ear infections**	*Citrullus lanatus (*thunb.) Matsum. & Nakai, *Cynanchum grandidieri* Liede & Meve
**Flue/Fever**	*Ocimum canum* Sims., *Croton geayi* Leandri
**Skin disorders**	*Lemuropisum edule* H. Perrier
**Post delivery care**	*Erythroxylum retusum* Baill. ex O.E. Schulz*, Salvadora angustifolia* Turill*, Loeseneriella rubiginosa* (H. Perrier) N. Hallé
**Toothache**	*Zanthoxylum tsihanimposa* H.Perrier*, Euphorbia tirucalli* L.
**Venereal infections**	*Cynodon dactylon* (L.) Pers.*, Euphorbia tirucalli* L.*, Blepharis calcitrapa* Benoist
**Respiratory system disorders**	*Cynanchum perrieri* Choux*, Indigofera compressa* Lam.
**Malaria**	*Cajanus cajan* (L.) Millsp., *Indigofera tinctoria* L.
**Sprains**	*Aloe divaricata* A.Berger*, Delonix floribunda* (Baill.) Capuron
**New born care**	*Coffea grevei* Drake ex A. Chev*, Pentatropis nivalis subsp. madagascariensis* (Decne.) Liede & Meve
**Circulatory system disorders**	*Opuntia* sp. (Raketamena)
**Woman genital hygiene**	*Ximenia perrieri* Cavaco & Keraudren*, Operculicarya decaryi* H. Perrier*, Cedrelopsis grevei* Baillon
**Cosmetic/Hair care**	*Ficus trichopoda* Baker*, Cedrelopsis grevei* Baill.
**Body tonic**	*Erythroxylum retusum* Baill. ex O.E. Schulz*, Neobeguea mahafaliensis* J.-F. Leroy*, Operculicarya decaryi* H. Perrier
**Nutritional disorders**	*Tamarindus indica* L.*, Adansonia za* Baill.*, Operculicarya decaryi* H. Perrier
**Livestock disease**	*Vigna unguiculata* (L.) Walp.

Apparently digestive system disorders (13%), wound and injury problems (12%) and post-delivery care for women (11%) represented the most prevalent health problems in the study area. The use of medicinal plants in cosmetic and genital care of women amounted to 8%, similar to plant use for ‘body tonic’ after hard physical work.

### Plant uses and knowledge patterns among households

Based on their socio-economic characteristics and the use intensity of forest products, the cluster analysis revealed two groups of households (Table 5). The well-off farmers represent households with a high number of livestock, off-farm activities and a higher education level. They use yam as a supplementary food, practice a more sustainable harvest technique and collect less wild yam tubers compared with the poorer farmers. The latter are characterized by lower household assets and off-farm activities. Farmers of this group collect more yam species and use their tubers as staple food.

**Table 5:** **Results of two step cluster and discriminant analysis of 250 interviewed rural households in the Mahafaly Region of SW-Madagascar**

Selected variables	Cluster group		Discriminant analysis		
	Well-off farmers	Less well-off farmers			
	Mean ± SD	Mean ± SD*	Wilks’ Lambda	Sig	Structure coefficients
**Education level**	1.03 ± 0.71	0.86 ± 0.69	0.986	0.068	0.116
**Agricultural harvest**	1.23 ± 0.42	0.52 ± 0.63	0.747	0.000**	0.574
**Households activities**	1.11 ± 0.71	0.26 ± 0.44	0.928	0.000**	0.274
**Family size**	7.35 ± 3.55	6.4 ± 3.20	0.982	0.037*	0.133
**Tropical livestock unit** ^**1)**^	12.53 ± 12.32	2.18 ± 5.40	0.746	0.000**	0.577
**Agricultural area**	2.86 ± 2.30	1.19 ± 1.60	0.945	0.000**	0.239
**Medicinal plants used**	27.77 ± 13.55	32.7 ± 14.30	0.974	0.011*	−0.162
**Number of medicinal uses**	13.87 ± 4.27	15.6 ± 3.60	0.976	0.016*	−0.153
**Diversity of medicinal plant use**	23.35 ± 2.12	25.92 ± 2.10	0.988	0.089	−0.108
**Wild yam species collected**	2.23 ± 2.71	3.17 ± 2.17	0.960	0.002**	−0.201
**Yam tubers harvested per collection event** ^**2)**^	6.72 ± 6.74	13.02 ± 10.33	0.908	0.000**	−0.314
**Frequency of collection**	2.35 ± 2.71	5.83 ± 5.23	0.886	0.000**	−0.354
**Sale**	3.95 ± 11.09	17.03 ± 24.12	0.920	0.000**	−0.291
**Collection period**	2.40 ± 2.29	13.78 ± 2.79	0.943	0.000**	−0.243
**Use of wild yams**	1.73 ± 0.44	1.49 ± 0.50	0.948	0.000**	0.231
			Eigen Value = 1.026		
			Percentage variance = 50.41		

^1)^[38]^2)^Number of harvest holes per collection event, *significance level at p ≤ 0.05, **significance level at p ≤ 0.01.

Most of the socio-economic variables used for the cluster analysis were effective in discriminating the two defined household groups except for the education level and the diversity of medicinal plant use. Together the predictors accounted for 51% of the between-group variability. Based on the conclusions of Rach et al. that structure coefficients ≥ 0.30 indicate a strong discriminating power [39], households cluster groups were determined by the amount of agricultural harvest, livestock owned by household, and the frequency of wild yams collection. In contrast, the number of medicinal plants used and the use intensity of medicinal plants differed only slightly among the two groups.

### Plant uses and knowledge patterns among villages

Collection and use of forest plants differed between the littoral (Efoetse) and the plateau (the other three villages) which may be mainly explained by the lack of forest resources and wild yams in the coastal area. The number of medicinal plants and wild yam species used were higher on the plateau (Ampotake, Andremba, Itomboina, Miarintsoa), and the number of species collected was highest in Itomboina and Miarintsoa (Table 6). However, the collection frequency, period, and the amount of harvested wild yam were higher in Ampotake. This may be mainly due to the proximity of community based forests, where collection of forest products is not restricted. Itomboina and Miarintsoa are situated in the middle of the plateau, where different soil types (ferralitic, red sandy and calcareous soils) and forest habitats prevail, which may explain the high diversity in species collection by the informants. Knowledge, traditional uses and the number of species used differ significantly (P < 0.01) among villages. Overall, the knowledge and the uses of plants are higher in Ampotake than in the other villages. In Ampotake, Miarintsoa and Itomboina, similar medicinal plant species are used as indicated by the Jaccard similarity indices ranging between 0.68-0.7 (Table 7).

**Table 6:** **Descriptive statistics of variables (Mean ± SD) used in evaluating the knowledge and uses of wild yams and medicinal plants of the Mahafaly region in SW-Madagascar**

Variables	Ampotake (n = 50)	Andremba (n = 50)	Itomboina (n = 50)	Miarintsoa (n = 50)	Efoetse (n = 50)
Collection of wild yams (%):					
*D. alatipes*	92.16	80.3	80	42	0
*D. bemandry*	94.12	51.52	80	87.23	0
*D. fandra*	54.9	60.61	60	59.57	0
*D. ovinala*	76.47	62.12	64.44	46.81	0
*D. nako*	43.14	21.21	66.67	48.94	0
*D. soso*	7.84	39.39	46.67	21.28	0
Number of wild yams species collected	3.9 ± 1.1	3.9 ± 1.3	4.2 ± 1.4	4.9 ± 1.9	0
Frequency of wild yams collection^1)^	9.8 ± 5.7	5.1 ± 2.5	5.6 ± 2.9	5.7 ± 3.9	0
Period of collection (months/year)	5.7 ± 1.9	4.1 ± 1.9	4.2 ± 1.4	4.9 ± 1.9	0
Wild yams harvested^2)^	21 ± 9	12.8 ± 5.8	14.1 ± 5.6	13.1 ± 7.6	0
Unsustainable harvest technique (%)	89.6	81.5	89.5	78.6	-
Sustainable harvest technique (%)	10.4	18.5	10.5	21.4	-
Monthly income, from wild yams (US$)^3)^	5.5 ± 7.4	1.3 ± 3.5	2.0 ± 3.0	1.3 ± 2.5	0
Number of medicinal species used	43.5 ± 12	29.8 ± 11.8	36.6 ± 10	27.4 ± 12.4	18.4 ± 9.7
Diversity of medicinal plant use	33.5 ± 10.3	23.9 ± 8.6	32.2 ± 7.7	23.4 ± 10.2	14.7 ± 7.7
Number of medicinal uses	17.6 ± 3.1	14.4 ± 3.2	16.7 ± 1.9	12.6 ± 3.3	12.8 ± 4.6

^1)^Times per month; ^2)^Number of harvest holes per collection event; ^3)^US$ = 2422 Ariary, 9.07.2014.

**Table 7:** **Similarity among medicinal plant species usage in the studied villages (Jaccard similarity indices, 1 = similar) in the Mahafaly region of SW Madagascar**

	Ampotake	Andremba	Itomboina	Miarintsoa	Efoetse
Ampotake	1	0.59	0.7	0.68	0.54
Andremba	0.59	1	0.58	0.58	0.43
Itomboina	0.7	0.58	1	0.71	0.55
Miarintsoa	0.68	0.58	0.71	1	0.51
Efoetse	0.54	0.43	0.55	0.51	1

### Effects of socio-economic characteristics on the use and knowledge of plants

The number of livestock owned (TLU), education level, family size and agricultural harvest were significant predictors for the number of medicinal plants used and the frequency of yam collection. The TLU and the age of respondents significantly affected the collection of wild yams (P < 0.001; Table 8). In the study region, a high number of livestock owned is a sign of wealth. Households with a low TLU are characterized by higher yam collection intensities. For the number of medicinal plants used, the only significant predictor variables were family size and healer consultancy. The latter indicates how often a household asked a traditional healer for advice on appropriate medicinal plants. The higher the diversity of different household activities (salaried work, trading, artisanal), the more cash income is produced. Consequently, the households have the possibility to buy food during difficult seasons, and depend less on wild food collection. In addition, female respondents use and know more plants than men. Age did not affect the use and knowledge on medicinal plants, which is maybe due to the direct knowledge transfer within one household. In this study, 79% of the households did not report to consult a traditional healer in case of illness.

**Table 8:** **Generalized linear Model (GLM) showing the effect of selected independent variables on the number of medicinal plants used and the collection frequency of wild yam (n = 250) in rural villages of the Mahafaly region in SW-Madagascar**

Independent variable	Number of medicinal plants used			Frequency of yam collection (Frequency month- ^1^ )		
	B*	P	r	B	P	R
Education level	−0.087	.029	−0.083	−0.249	0.008	−0.118
Tropical livestock unit	−0.007	.038	−0.192	−0.460	0.000	−0.263
Agricultural harvest	−0.127	.002	−0.270	−0.251	0.012	−0.229
Age	0.002	.217	0.119	−0.014	0.000	−0.209
Family size	0.027	.001	0.119	0.056	0.003	0.092
Gender	0.125	.029	0.128	0.153	0.232	0.124
Healer consultancy	−0.472	.000	−0.380	-	-	-
Households activities	-	-	-	0.053	0.550	0.038

(*) Beta coefficient; (r) regression coefficient, (−) the variable was not included in the model.

## Discussion

### Characteristics of the interviewed households

The basic characteristics of the interviewed households correspond to the results of INSTAT [22] for SW Madagascar even though our survey indicated a higher education level. In Ampotake, the majority of the households heads (52%) are illiterate, which reflects the percentage of the non-educated people in the rural area in this region. The average land size per household (2.2 ha) corresponds to the respective value in Mozambique [40]. In this study, we used off-farm activities to determine the different cash income sources and diversification level of households based on the assumption that higher diversification leads to higher income [41, 42].

### Traditional knowledge and usage of wild yams

Among the six species of wild yam recorded, only *D. alatipes* and *D. bemandry* were frequently harvested by local people to substitute for staple food. This is comparable to the collection of wild yam species in the dry forest of NW-Madagascar [43]. Mavengahama et al. [44] recorded a similar importance of wild yam collection for rural livelihoods in South Africa, where wild vegetable are of high importance in supplementing staple food diets based on maize, sorghum (*Sorghum bicolor* Moench.), and millet (*Pennisetum glaucum* L.).

In our study, the collection intensity of wild yams depended not only on the availability of the species, but also on the taste of the yam tubers. For Malagasy yams, the preference in taste was analysed by Jeannoda et al. [14] who observed a significant correlation (P < 0.001) between the preference and the sensitivity to saccharose. Polycarp et al. [45] stated that the high level of carbohydrate and energy with appreciable levels of minerals makes yam a very nutritious source of food. Bhandari et al. [46] found that the nutritional composition of selected wild yams in Nepal was similar to those reported for cultivated species of yam. When analyzing the nutritional value of Malagasy yam germplasm, including those of wild species, Jeannoda et al. [14] determined high contents of calcium in *Dioscorea ovinala,* which makes some wild yams physiologically important.

However, a decline in the availability of wild yams was already reported by the respondents of our study who are forced to increase the search radius for tuber harvests. One main reason for the decline in this essential resource securing local livelihood strategies against drought related hunger risks may be the exploitative harvesting methods used by the majority of the collectors in the Mahafaly region, which hampers the regeneration of the species. In contrast, Ackermann [43], who conducted a study in the NW-Madagascar reported that traditional people try to harvest the tubers carefully to guarantee the survival of the plant stand. In our study only 15% of the household took care of the regeneration of the lianas. While the sale of wild yam tubers provides valuable cash income for many households it may also be one of the causes for its overexploitation and increasingly threatened existence [47]. About 20% of the harvested tubers per households are sold on local markets.

### Traditional knowledge and usage of medicinal plants

The majority of the medicinal plants used by the local people belong to the Fabaceae, Apocynaceae and Euphorbiaceae. In contrast to yams, none of the interviewed households was selling medicinal plants. Local people complained that some species are nowadays hard to find, which was confirmed by our field observation. Hamilton [48] stated that globally 4,160 to 10,000 medicinal plants are endangered by habitat losses or overexploitation in areas where rural families traditionally collected them. The present study shows that the most popular plants with high use values, such as *Aloe divaricata*, *Erythroxylum retusum*, *Cedrelopsis grevei*, *Neobeguea mahafaliensis*, *Salvadora angustifolia* and *Croton geayi* are native species collected from forest habitats. This shows that the wild habitats are important for local communities in terms of basic needs. Beltrán-Rodríguez et al. [49] also pointed to the importance of wild habitats for peoples’ livelihood in a rural community of Mexico and found a greater diversity of plant uses in wild habitats than in managed environments.

Some plants are less frequently used, which does not decrease their importance since most of them are needed for very specific therapeutic purposes. The increasing scarcity of such plants may also enhance the loss of traditional knowledge about the medicinal uses [50, 51]. On the other hand there are cultivated species such as *Tamarindus indica* and *Sclerocarya birrea* subsp. *caffra*, *Citrullus lanatus* and *Ziziphus spina-christi,* which are nowadays used more intensively for medicinal purposes.

Different parts of the same plant are used for different purposes or by different population groups. Sometimes, a specific plant part is used for children and another part of the same plant for adults to treat a disease such as in the case of *Aloe divaricate*. The use of plant roots as traditional remedies is often problematic as it prevents plant regeneration [52]. Muthu et al. [53] reported that the choice of plant species most used by people depended largely on the type of diseases treated. In our study, digestive disorders, post-delivery care, body injuries and wounds were the most frequently mentioned diseases. This is comparable to similar studies conducted in Africa [54, 55] China [56] and in Colombia [57], where digestive disorders were most frequently treated by medicinal plants. Compared to other developing countries, where sexually transmitted infections are most commonly treated with herbal medicines [58] this category was rarely cited in our study. Except for venereal diseases which are treated using a combination of different species [59, 60] the majority of plant species utilized had a single therapeutic use.

Some of the recorded medicinal plants in Madagascar are already pharmaceutically analysed and the active ingredients confirm traditional therapeutic uses. For example*, Koehneria madagascariensis* has a large and strong antimicrobial activity [61]. *Hernandia voyronii*[62] is known for its antimalarial active substances, *Neobeguea mahafaliensis* and *Cedrelopsis greveii* for effectiveness against cardiovascular diseases [63]. Although the World Health Organization (WHO) reported that 60-70% of Madagascar inhabitants have ready access to primary health care [64], accessibility of effective modern medicines is still a challenge for the local population in the Mahafaly region and they thus make use of native plants for alternative treatment.

### Effects of socio-economic conditions on the use of wild yams and medicinal plants

Our study revealed that the collected quantities and qualities of plants vary greatly between households. Very poor and poor farmers consume and sale more yams and have higher knowledge on traditional usages of medicinal plants than well-off or “rich” individuals. Households with lacking off-farm income collect and consume more frequently wild yams than households with regular off-farm income. In addition, the regression results revealed, that households with more cropland and higher crop harvest collect less forest products. This was also confirmed by Reddy and Chakravarty [65] in India. Variables showing the collection and consumption of wild yams (P < 0.01) were important discriminators for household groups in contrast to the variables on the use of medicinal plants (P < 0.05).

The use of forest products was significantly higher in villages near forests, where wild yams and medicinal plants are more readily available. This confirms findings of Banana and Turiho-Habwe [66] in Uganda and Kerapeletswe and Lovett [67] in Botswana, where the dependency on the forests for food supply decreased rapidly with an increasing distance of the respondent’s home from the forests. Furthermore, poor market access may increase the importance of forest products to sustain people’s livelihood [68].

The number of livestock owned by the household, education level, agricultural harvest and family size affected the collection of wild yams and the usage of medicinal plants. Livestock and off farm activities determine the wealth condition of the household in this region and were negatively correlated with the use of wild yams and medicinal plants. However, we cannot generalize these findings as with time and location the direction of the relationship may change [69]. Socio-cultural factors are of higher importance for the use of medicinal plants than for the collection of wild yams. In contrast to other findings [49] female respondents use more plant species than males. The use of medicinal plants is the basic health care for the majority of the households and the knowledge about their use was maybe shared over generations, which might explain, that there is no significant influence of informant age on the collection intensity of medicinal plants. In the study of Kirstin [70] on the usage of Budongo’s forest products, the use of wild food such as *Dioscorea* spp. increased with age, whereas young village people focused on the use of fruits and wild game because of their higher income potential. This might also be true for our study region, were younger farmers predominate in collecting wild yams for sale.

Overall, this study indicates that a household’s wealth status affects the traditional knowledge and use intensity of forest products, which confirms previous studies [49, 71, 72]. The World Resources Institute [4] reported that families facing poverty, sickness, drought, wars and economic crisis depend to a higher degree on the collection of wild resources. Although, our study focused only on medicinal plants and wild yams as forest products, the rate of change in social and economic attributes of rural households is likely proportional to the rate of change in resource use [73]. Therefore, whatsoever the products extracted, a household’s socio-economic dynamics ultimately drives its ability to use village forest resources.

## Conclusions

Our results revealed that wild yams play an important role in local food security in the Mahafaly region, especially for poor farmers. On the other hand, medicinal plants are a primary source of health care for the majority of local people in SW-Madagascar and the results of this study can help to identify the most useful plant species and their importance for the local people. In many rural areas of developing countries, common property resource management plans may allow to combine poverty reduction and biodiversity conservation. In our study region the forest patches around the Tsimanampetsotsa National Park are managed by local communities. Our results indicate the influence of socio-economic household characteristics on the use of forest products and its intensity, which should be considered in future management plans for local and regional forest conservation.
